# Assessing potential risk factors for metamizole-induced leukopenia

**DOI:** 10.1177/20420986241275255

**Published:** 2024-09-18

**Authors:** Birgit Brüne, Sarah Sonderer, Maria Bösing, Simona Hübner, Kanchan Dongre, Selina Späni, Andreas Holboro, Jörg D. Leuppi, Anne B. Leuppi-Taegtmeyer

**Affiliations:** University Center of Internal Medicine, Cantonal Hospital Baselland, Liestal, Switzerland; University Center of Internal Medicine, Cantonal Hospital Baselland, Liestal, Switzerland; University of Basel, Basel, Switzerland; University Center of Internal Medicine, Cantonal Hospital Baselland, Liestal, Switzerland; University of Basel, Basel, Switzerland; University Center of Internal Medicine, Cantonal Hospital Baselland, Liestal, Switzerland; Department of Patient Safety, Medical Directorate, University Hospital Basel, Basel, Switzerland; Clinical Pharmacology and Clinical Pharmacy, Hospital Pharmacy, Cantonal Hospital Baselland, Liestal, Switzerland; University of Basel, Basel, Switzerland; Regional Blood Transfusion Centre, Swiss Red Cross, Basel, Switzerland; Division of Hematology, University Hospital Basel and University of Basel, Basel, Switzerland; University Center of Internal Medicine, Cantonal Hospital Baselland, Liestal, Switzerland; University of Basel, Basel, Switzerland; University of Basel, Spitalstrasse 22, Basel 4031, Switzerland; Department of Patient Safety, Medical Directorate, University Hospital Basel, Basel, Switzerland; Clinical Pharmacology and Clinical Pharmacy, Hospital Pharmacy, Cantonal Hospital Baselland, Liestal, Switzerland

**Keywords:** agranulocytosis, dipyrone, leukopenia, metamizole, risk factors

## Abstract

**Background:**

Metamizole is a non-opioid analgesic agent that can rarely cause agranulocytosis, a severe form of leukopenia.

**Objectives:**

The aim of this study was to assess previously identified potential risk factors for the development of metamizole-induced leukopenia.

**Design:**

A retrospective, observational, matched case-control study was performed in a single-center setting.

**Methods:**

Patients who developed leukopenia in the setting of metamizole therapy were included as cases and matched 1:3 on the basis of age and sex to control patients who did not develop leukopenia when treated with metamizole. The data were obtained from the medical records of patients hospitalized at Cantonal Hospital Baselland between 2015 and 2020. Univariate and multivariate analyses were performed.

**Results:**

Eighty-six cases and 258 matched controls aged between 18 and 102 years were included. Fifty-seven percent were female. Previous leukopenic episodes (odds ratio (OR): 4.02, 95% CI: 1.95–8.28, *p* < 0.001) and a history of penicillin allergy (OR: 2.49, 95% CI: 1.03–6.03, *p* = 0.044) were found to be independent risk factors for metamizole-induced leukopenia.

**Conclusion:**

A history of previous leukopenic episodes and a history of penicillin allergy were confirmed as risk factors for metamizole-induced leukopenia. In our opinion, metamizole should be avoided in patients with these risk factors.

## Plain language summary

Assessing potential risk factors for metamizole-induced leukopenia

Background: Metamizole is a medication used to treat pain and fever. It carries a risk of developing the side effect of a low white blood cell count (leukopenia). Researchers have identified certain risk factors which predispose some, but not all, people to develop this side effect. We undertook this study to examine these risk factors in more detail.

Method: We compared hospitalized patients treated with metamizole who developed leukopenia, with similar hospitalized patients who did not develop this side effect.

Results: It was observed that patients were more likely to develop leukopenia under metamizole therapy if they:

- had previous episodes of leukopenia

- were under cytostatic/immunosuppressive therapy (for example drugs used to treat cancer or autoimmune conditions)

- had a history of penicillin allergy.

Conclusion: These findings will help in identifying people who are at risk of developing this serious side effect, so that they can be given a medication for pain or fever that suits them better.

## Introduction

Metamizole is an aminopyrine and non-acid non-opioid analgesic that was first introduced in Germany in 1922.^1^ It is used to treat fever, postoperative pain, cancer-related pain, or pain caused by injuries.^2^ However, the exact mechanism of action is still unclear. In Switzerland, metamizole requires a prescription,^1,3^ while in other countries such as Turkey and Israel, it is available over the counter.^1^ The licensed indication in Switzerland is “severe pain and high fever that do not respond to other measures.”^3^ According to the drug report of Swiss health insurance from 2020, metamizole purchases in Switzerland increased by 26% between 2016 and 2019.^4^

Metamizole is not licensed in several countries (e.g. Australia, USA, Japan, and Sweden) due to its rare but potentially fatal adverse drug reaction (ADR), agranulocytosis.^5^ The incidence of agranulocytosis is estimated to be between 1 in 3000 users/year^6^ and 1 in 1 million users/week.^5,7^ A recent study examining German statutory health insurance data found the risk for drug-induced agranulocytosis and neutropenia after metamizole prescription to be 1:1602 patients.^8^ Agranulocytosis has been reported to occur within a few hours of exposure,^9^ while other reports describe its occurrence 28 days or more post intake.^10,11^ In an analysis of 858 metamizole-associated hematological ADRs, more than half (52%) occurred within 7 days of exposure to metamizole.^5^ The mechanism by which metamizole causes blood disorders has not yet been fully elucidated. Available data suggest an immunological process, as well as direct toxicity toward the progenitor cells in the bone marrow.^12^

The ADR metamizole-induced leukopenia is a pathological decrease in total leukocyte count to below the normal range during metamizole therapy. Agranulocytosis is a severe type of leukopenia characterized by an absolute neutrophil count of <0.5 × 10^9^/L.^9^ Potential risk factors for the development of leukopenia related to metamizole therapy have previously been identified in retrospective studies and include previous leukopenic episodes, a history of allergies or drug hypersensitivity, autoimmune diseases, hepatitis, and concomitant treatment with other drugs (especially methotrexate).^9,13^ The aim of this study was, therefore, to assess these findings in an independent data set in a similar patient population in Switzerland.

## Methods

### Study design

This retrospective, observational, matched case-control study analyzed data from the medical records of hospitalized patients at Cantonal Hospital Baselland (KSBL) between 2015 and 2020. The reporting of this study conforms to the Strengthening the Reporting of Observational Studies in Epidemiology (STROBE) statement^14^ (see Supplemental Material for checklist).

### Patients

Patients aged over 18 years and hospitalized between 2015 and 2020 who had not denied consent for their health-related data to be analyzed for research purposes were included in the study. During the study period, medical records were created in the software program Polypoint (POLYPOINT AG, Gümligen, Switzerland) and stored in an electronic archive (health-engine, the i-engineers, Zürich, Switzerland) at KSBL. Discharge reports were a main source of information as they included detailed patient summaries and were written by at least two physicians (one physician in postgraduate training and one senior physician). The discharge reports were characterized by high validity and reliability. They were available as PDFs, meaning that they could not be altered after having been signed.

Cases were patients diagnosed with metamizole-induced leukopenia. They were identified from the medical records using the International Classification of Diseases (ICD) diagnostic code D70.1 for ‘Drug-induced agranulocytosis and neutropenia’ as there is no specific ICD code for drug-induced leukopenia.^15^ Cases with an ICD code D70.1 documented to be metamizole- induced were included in the study if laboratory measurements were confirmatory (leukocyte count below 3.9 × 10^9^/L and/or neutrophil count <1.5 × 10^9^/L as per local laboratory definition). The hospital laboratory has had continuous ISO 15189 accreditation since 2001.

Controls were patients who received metamizole without developing hematological complications. In order to find controls, internal billing data were used to identify all metamizole prescriptions issued at our institution between 2015 and 2020. In the next step, we matched the cases with the controls according to age, sex, and year of hospitalization. We then checked the discharge reports, medication charts, and laboratory values of the selected controls in order to confirm metamizole intake and rule out metamizole-induced leukopenia. Matching controls were selected randomly and were replaced if the inclusion criteria were not met.

### Sample size considerations

**Figure 1.**
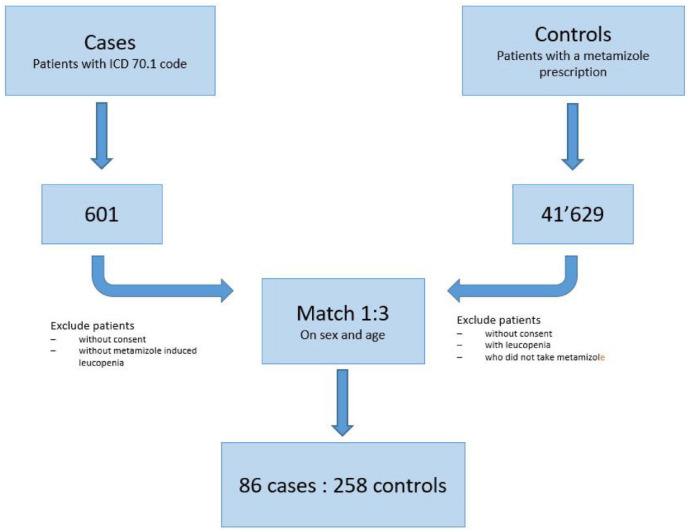
Flow diagram showing case and control selection.

In 2017, a study was published using data from a similar-sized hospital over a slightly longer period of time (2005–2013).^13^ It examined 57 cases of metamizole-induced leukopenia and 139 matched controls. In order to reduce confounding and increase the power of the current study, we performed a 1:3 (cases:controls) matching for sex and age as well as year of hospital admission on a matched pair basis.^16^ Cases could be matched to controls who were up to 6 years older or younger. Figure 1 shows the number of patient data sets examined and ultimately included in the study.

### Data collection and statistical analysis

Supplemental Table S1 lists the parameters that were collected for cases and controls. Data were collated in a Microsoft Excel spreadsheet. The statistical computing software R (version 4.0.2, R Foundation for Statistical Computing, Vienna, Austria) was used to perform descriptive, univariable, and multivariable analyses. For descriptive statistics of continuous variables, the mean, standard deviation, median, interquartile range, and the number of missing values were determined for the cases and the controls. For categorical variables, the absolute number and percentages within cases and controls were calculated. Comparisons were performed using the Chi-square test for categorical data and the Mann–Whitney *U* test for non-normally distributed, continuous data. Univariable and multivariable logistic regression analyses were performed, and results were expressed as odds ratios (ORs) and adjusted ORs, respectively. The risk factor was considered as significant if the *p* value was ⩽0.05, and the OR was >1. Variables that were significantly associated with leukopenia in the univariable analysis were included in the multivariable logistic regression analysis. For the multivariable model, multicollinearity was assessed using a correlation matrix (Pearson’s rho). Missing data were reported as missing, and no imputation was performed.

## Results

### Demographic data

A total of 344 patients (86 cases and 258 controls) were included in the study. Table 1 shows the demographic data and length of hospital stay of the study population.

**Table 1.** Patient demographics.

Characteristic	Cases	Controls	*p* Value
Number	86	258	
Age (y), mean ± SD	65.81 ± 19.6	63.73 ± 19.7	0.16*
Median (range)	68.0 (18–98)	66.0 (19–102)	
Female, *n* (%)	49 (57)	147 (57)	1.00**
Length of hospital stay (days), median (IQR)	8 (9)	3 (7)	<0.0001*
Median time between hospital admission and diagnosis of ADR (days; range)	2 (−6 to 34)	Not applicable	
Number of patients diagnosed with ADR on day of admission or before admission (%)	22 (26)	Not applicable	

* Mann–Whitney *U* test.

** Chi-square test.

ADR, adverse drug reaction; IQR, interquartile range.

Eleven patients with metamizole-induced leukopenia and one patient without received concomitant cytostatic agents. These are given in Supplemental Table S2.

### Adverse drug reaction

The distribution of the specific hematological ADRs among cases is shown in Tables 2 and 3, while the actual laboratory values on the day of diagnosis are summarized in Table 4. Thirty-six percent of patients had an isolated leukopenia, while 44% (the majority) had a bicytopenia and 19% had a pancytopenia. Of the patients with bicytopenia, leukopenia and anemia (82%) were more common than leukopenia and thrombocytopenia (18%).

**Table 2.** Distribution of hematological adverse drug reactions among cases.

Type of cytopenia	Number of cases (%)
Leukopenia alone (leukocyte count <3.9 × 10^9^/L)	31 (36)
Bicytopenia	38 (44)
Leukopenia + anemia (hemoglobin <120 g/L)	31* (82)
Leukopenia + thrombocytopenia (platelet count <150 × 10^9^/L)	7 (18)
Pancytopenia (leukopenia + anemia + thrombocytopenia)	16 (19)
Leukopenia with unknown hemoglobin and platelet count values	1 (1)

* One patient had anemia and a normal leukocyte count due to lymphocytosis and hypereosinophilia; however, the neutrophil count was 0.8 × 10^9^/L.

**Table 3.** Characteristics of neutropenia.

Severity of neutropenia (*n* = 65 patients with a neutrophil count (76%))	Number of cases (%)
No neutropenia (absolute neutrophil count >1.5 × 10^9^/L)	13 (20)
Mild or moderate neutropenia (absolute neutrophil count 0.5–1.5 × 10^9^/L)	31 (48)
Severe neutropenia (absolute neutrophil count <0.5 × 10^9^/L)	21 (32)

**Table 4.** Laboratory parameters on the day of cases’ ADR diagnosis.

Blood cells (normal range)	Number of measurements	Mean ± SD	Median (range)
Leukocytes (3.9–10.2 × 10^9^/L)	86	2.56 ± 0.93	2.80 (0.4–4.4)
Neutrophils (1.5–6.7 × 10^9^/L)	65	0.99 ± 0.72	1.1 (0.00–2.6)
Hemoglobin (120–160 g/L)	85	116 ± 25.74	115 (57–260)
Platelets (150–450 × 10^9^/L)	85	204 ± 81.69	198 (15–437)

### Potential risk factors for metamizole-induced leukopenia

Table 5 shows the results of the univariable analysis of the postulated risk factors. Correlations between predictor variables were low (*r* < 0.16).

**Table 5.** Frequency of postulated risk factors and their OR for metamizole-induced leukopenia.

Postulated risk factor or confounder	Cases	Controls	OR	95% CI	*p* Value*
History of allergy (%)	27 (31)	57 (22)	1.61	0.94–2.78	0.084
Medication allergies (%)	20 (23)	40 (16)	1.65	0.90–3.02	0.10
Penicillin (%)	11 (13)	15 (6)	2.38	1.05–5.39	0.039
Non-steroidal anti-inflammatory drugs (%)	4 (5)	6 (2)	2.05	0.56–7.44	0.276
Non-medication allergies (%)	9 (10)	23 (9)	1.09	0.49–2.43	0.84
Infections (%)	28 (33)	50 (19)	2.01	1.16–3.47	0.012
Bacterial infections (%)	22 (26)	40 (16)	1.87	1.04–3.38	0.037
Viral infections other than hepatitis C (%)	7 (8)	11 (4)	0.99	0.75–5.31	0.17
Hepatitis C infection (%)	3 (4)	2 (1)	4.63	0.76–28.16	0.097
Previous leukopenic episodes (%)	23 (27)	18 (7)	4.87	2.48–9.57	<0.001
Concomitant cytostatic agents (all; %)	11 (13)	1 (0.4)	37.69	4.79–296.44	<0.001
Concomitant low-dose antimetabolites (methotrexate or azathioprine; %)	4 (5)	1 (0.4)	12.54	1.38–113.75	0.025
Length of hospital stay (days; median [IQR])	8 [5–14]	3 [1–8]	1.02	1.00–1.05	0.032

Length of hospital stay was also analyzed as a possible confounder. Figures are numbers of patients (%) unless otherwise stated. (Some patients had multiple allergies or infections which is why the figures do not add up to the total in the first and sixth rows.)

* Univariable logistic regression.

IQR, interquartile range; OR, odds ratio.

Patients with allergies were analyzed in more detail. In the study population, 84 patients (24%) had at least one self-reported or documented allergy. A total of 135 different allergies were reported. Patients had between one and four different allergies. The allergies were categorized as medication allergies and non-medication allergies. The non-medication allergies included allergies to pollen, animal fur, food, and other allergens. Overall, medication allergies were more common than non-medication allergies. Penicillin- and non-steroidal anti-inflammatory drugs allergies were the most common medication allergies (Table 5).

Table 6 shows the results of the multivariable analysis, which was performed using the parameters that showed a significant association with the development of metamizole-induced leukopenia in the univariable analysis. Because cytostatic agents commonly cause leukopenia, they are a potential source of bias, so we did not include patients who received them (*n* = 12, Table 5) in this analysis. The results of the multivariable analysis showed a history of penicillin allergy and previous leukopenic episodes to be independent risk factors for leukopenia associated with metamizole therapy. These risk factors were independent of length of hospital stay, for which the statistical model was adjusted.

**Table 6.** Multivariable analysis of risk factors for metamizole-induced leukopenia, adjusted for length of hospital stay.

Postulated risk factor or confounder	OR	CI	*p* Value*
History of penicillin allergy	2.49	1.03–6.03	0.044
Infections	1.76	0.96–3.24	0.067
Previous leukopenic episodes	4.02	1.95–8.28	< 0.001
Length of hospital stay	1.02	1.00–1.04	0.058

* Multivariable logistic regression.

CI, confidence interval; OR, odds ratio.

## Discussion

The aim of this study was to assess previously identified potential risk factors for the development of leukopenia associated with metamizole in a real-life study and so to provide more information on the safe use of metamizole. We found that previous leukopenic episodes and a history of penicillin allergy were independent risk factors. Due to inherent bias, the concomitant use of cytostatic agents (including low-dose antimetabolites used for immunosuppression) was not investigated as an independent risk factor. A history of allergies in general and infection with hepatitis C, as found by Blaser et al.,^13^ were not confirmed as risk factors.

### Demographic data

The population studied here was comparable to other studies of leukopenia and neutropenia associated with metamizole use in terms of gender distribution (57% female in the present study compared to 50–76% reported in other studies)^8,9,12,13^ Our patient population had a higher median age than other studies: 68 years compared to 44–58 years.^9,12,13^

### Adverse drug reaction

Most studies to date have investigated severe forms of metamizole-induced leukopenia, namely, neutropenia or agranulocytosis.^8,9,12^ Like another study conducted in the same geographic region,^13^ we elected to study metamizole-induced leukopenia because in our experience, it is a clinically relevant ADR, which when detected early can be managed by stopping metamizole and so prevent further decline in the neutrophil count. Therefore, although our study did include patients with agranulocytosis (32%), direct comparisons to the studies mentioned above^8,9,12^ cannot be made.

### Potential risk factors for metamizole-induced leukopenia

#### Previous leukopenic episodes

Blaser’s study highlighted previous leukopenic episodes as a risk factor for the development of metamizole-induced leukopenia^13^ We could confirm this in both univariable and multivariable analyses, indicating that this is an independent risk factor. Neither the present study nor the study by Blaser et al. reported the likely cause of the previous leukopenic episodes. However, regardless of the cause, two separate studies in two different populations spanning different time periods have found previous leukopenic episodes to be associated with metamizole-induced leukopenia. In our opinion, this implies that metamizole should be avoided in patients with previous leukopenic episodes.

### Concomitant cytostatic agents

In the univariable comparison, the odds of developing metamizole-induced leukopenia were 38 times higher in patients with cytostatic agents compared to those without. This corroborates the findings of previous studies.^9,13^ Furthermore, patients receiving low doses of antimetabolites (methotrexate or azathioprine) were also at increased risk of developing leukopenia with concomitant metamizole (OR 12.5). Due to the small sample size (*n* = 4 cases and one control), multivariable comparison was not feasible. However, it is not possible to ascertain causality in this situation, as cytostatic agents commonly cause hemotoxicity. Nevertheless, in our opinion, a pharmacodynamic drug–drug interaction can be postulated. To prove this, a further study of patients receiving cytostatic agents with and without metamizole needs to be performed.

In our opinion, despite not being able to prove causality, metamizole should be avoided in patients with concomitant cytostatic agents, even at low doses, particularly in patients receiving methotrexate.

### Allergies

A history of allergies in general could not be confirmed as a significant, independent predictor in our study. The presence of a penicillin allergy was more common among cases (13%) than among controls (6%), as also observed in the study by Blaser et al.^13^ (beta-lactam allergy overall 21% vs 10%). A history of penicillin allergy was furthermore found to be an independent risk factor for the development of leukopenia during metamizole therapy. Postulated mechanisms for this observation include the presence of multiple drug hypersensitivity^17^ and manifestation of different drug allergies during concomitant viral infection.^18^

Drug allergy was not confirmed as a risk factor for metamizole-associated neutropenia in a smaller study of 48 cases and 39 unmatched controls by Rudin et al.^12^ One reason for the discrepant findings is likely the different study endpoint and another reason may be the relatively small sample size and unmatched design (total 87 patients, compared to the 344 studied here). As detailed information about the reported allergies such as their manifestation and diagnostic workup was not available, no conclusions about the underlying mechanism for the observed association can be drawn.

### Infections

An increased risk for developing ADRs to a variety of drugs has been shown for viral infections such as HIV, hepatitis C, Epstein–Barr virus, herpes simplex virus, human herpesvirus, and cytomegalovirus infection.^19–22^ The exact mechanisms by which viral infections cause an increased risk of ADR are not fully known. Reduced immune tolerance, increased antigenicity, and altered drug metabolism have been proposed as the likely causes.^18,23^ In addition, the chronic viral infection itself may be associated with an altered blood count, including leukopenia^24^ The risk factor of hepatitis C identified by Blaser et al. could not be confirmed in the present study; however, this may be due to the small sample size (five patients known to be hepatitis C positive).

### Genetics

Although we did not carry out genetic testing in our study, potential genetic risk factors could play a role in identifying patients who should avoid metamizole.

Despite genetic predisposition having been implicated as a risk factor for the development of agranulocytosis under metamizole, there has been no conclusive evidence in the scientific literature to date. This may be due to the relatively small sample sizes of the studies, differences in populations, and the heterogeneity in the causes and phenotypes of metamizole-induced agranulocytosis. Potential genetic loci that have so far been identified are two candidate loci on chromosome 9 (one of which located in the *SVEP1* gene previously implicated in hematopoiesis),^25^ NAT2, CYP2C9, and CYP2C19 gene polymorphisms^26^ and the presence of HLA24.^27^ Further studies are needed to gain additional insights into genetic predisposition to and the mechanisms underlying metamizole-induced leukopenia.

### Limitations

Due to the retrospective nature of this study, missing data and incomplete data are a limitation. Another limitation is posed by the relatively small number of study subjects due to the rarity of this ADR. While the cases included in the study were most likely caused by metamizole, as in all pharmacovigilance cases, there cannot be complete certainty. Furthermore, it was not possible to retrospectively ascertain the exact details of metamizole therapies in terms of start and stop dates, total cumulative dose, outcome of rechallenges (if any) and treatment duration, total amount of concomitant medication, all allergies, and infections. Other than cytostatic agents, potential further leukopenia-causing agents could not be accounted for. The case and control groups differed in terms of average length of hospital stay, which was probably due to the leukopenia itself, or might have had a non-medical cause such as need to wait for place in a rehabilitation center or nursing home. Adjustment of the multivariable model for length of hospital stay, however, did not alter the associations with the examined risk factors (penicillin allergy and previous leukopenic episodes). Lastly, no genetic studies were performed in our study.

### Generalizability

Our study was carried out in a population hospitalized in a Swiss regional hospital, which also includes a university clinic of internal medicine. Our study findings were in keeping with a previous study performed in a university hospital in the same geographical region of Switzerland. We assess our results as being generalizable to other Swiss hospitals and clinics where metamizole is given for fever and pain management.

## Conclusion

In this real-life study, we confirmed the findings of earlier studies that previous leukopenic episodes are independently associated with a higher risk for developing leukopenia during metamizole therapy. Concurrent penicillin allergy was also an additional, independent risk factor. These readily available parameters should be routinely considered when prescribing metamizole to patients. The findings should form the basis for future research to develop risk scores and integrate these into clinical decision support systems. Prescribing physicians could then be automatically alerted when attempting to prescribe metamizole to patients with a high-risk constellation for developing leukopenia.

## Supplemental Material

sj-docx-1-taw-10.1177_20420986241275255 – Supplemental material for Assessing potential risk factors for metamizole-induced leukopenia Supplemental material, sj-docx-1-taw-10.1177_20420986241275255 for Assessing potential risk factors for metamizole-induced leukopenia by Birgit Brüne, Sarah Sonderer, Maria Bösing, Simona Hübner, Kanchan Dongre, Selina Späni, Andreas Holboro, Jörg D. Leuppi and Anne B. Leuppi-Taegtmeyer in Therapeutic Advances in Drug Safety

## Declarations

**Ethics approval and consent to participate:** The study complies with the regulatory requirements of the Swiss Health Research Act and Health Research Ordinance. The study was approved by the ‘Ethikkommission Nordwest- und Zentralschweiz’ (project-ID 2021-00284). As data were analyzed anonymously, no individual patient informed consent was required; however, all patients had previously given their prior written, general consent for their anonymized data to be analyzed for research purposes.

**Consent for publication:** Not applicable.

**Competing interests:** The authors declare that they have no conflict of interest in relation to this work. JL has been supported by grants from the Swiss National Science Foundation (SNF 160072 and 185592) as well as by the Swiss Personalized Health Network (SPHN 2018DR108). He has also received unrestricted grants from AstraZeneca AG Switzerland, Boehringer Ingelheim GmbH Switzerland, GSK AG Switzerland, OM Pharma AG Switzerland, and Sanofi AG Switzerland.

**Availability of data and materials:** The authors confirm that the data supporting the findings of this study are available within the article.
